# Prevalence and Correlates of Vitamin D Deficiency in Children Aged Less than Two Years: A Cross-Sectional Study from Aseer Region, Southwestern Saudi Arabia

**DOI:** 10.3390/healthcare10061064

**Published:** 2022-06-08

**Authors:** Saleh M. Al-Qahtani, Ayed A. Shati, Youssef A. Alqahtani, Samy A. Dawood, Aesha F. Siddiqui, Mohamed Samir A. Zaki, Shamsun N. Khalil

**Affiliations:** 1 Department of Child Health, College of Medicine, King Khalid University, Abha P.O. Box 62529, Saudi Arabia; smuadi@kku.edu.sa (S.M.A.-Q.); yal-qahtani@kku.edu.sa (Y.A.A.); samyshorbagy8@hotmail.com (S.A.D.); 2 Department of Family and Community Medicine, College of Medicine, King Khalid University, Abha P.O. Box 62529, Saudi Arabia; draeshasiddiqui@gmail.com (A.F.S.); shamsun203@gmail.com (S.N.K.); 3 Department of Anatomy, College of Medicine, King Khalid University, Abha P.O. Box 62529, Saudi Arabia; mszaki@kku.edu.sa; 4 Department of Histology and Cell Biology, College of Medicine, Zagazig University, Zagazig P.O. Box 31527, Egypt

**Keywords:** vitamin d deficiency, children, sunlight exposure, Saudi Arabia

## Abstract

*Background:* Vitamin D is an essential nutrient for bone growth, mineralization, and other metabolic processes in the human body. Hence, insufficiency or deficiency of this vitamin can have long-term effects, particularly for children. *Objectives:* The aims of this study were to determine the prevalence of vitamin D deficiency in children up to 2 years of age and investigate the independent predictors of vitamin D deficiency. *Methodology:* This cross-sectional study was conducted among 484 children aged up to two years who were admitted to the hospital for the treatment of any acute condition from January to November 2021. Serum 25(OH)D was used to determine the level of vitamin D. The serum 25(OH)D was categorized into 3 groups: Sufficiency (>30 ng/mL), insufficiency (20–30 ng/mL), and the deficiency (<20 ng/mL). *Results:* Overall, vitamin D deficiency was observed in 70.5% of the children, of whom 45.9% had insufficient levels, and one-fourth (24.6%) showed deficiency. The children aged 2–12 months (infants) were more likely to be vitamin deficient compared to children aged 12 months and above. The children who lived in urban areas had a threefold increased risk of vitamin D deficiency (aOR = 3.0, 95% CI 1.78–5.08). The children who were exposed to sunlight for less than 3 days per week experienced a higher risk of developing vitamin D deficiency (aOR = 4.17, 95% CI 2.04–10.88). Children who had received only breast milk were more than two times more likely to experience vitamin D deficiency (aOR = 2.42, 95% CI 1.12–5.23) compared to their counterparts. *Conclusion:* Our study reveals a high prevalence of vitamin D deficiency among children aged up to two years. Infants, urban dwellers, only breastfed, and exposure to sunlight for less than three days per week were identified to be the independent risk factors for vitamin D deficiency. The results of this work call for enhancing awareness to ensure adequate levels of vitamin D for better health of the children in this region of Saudi Arabia.

## 1. Introduction

Vitamin D is a lipid-soluble vitamin that is known to play an important role in bone metabolism through the regulation of calcium and phosphate homeostasis. Thus, insufficiency or deficiency of this vitamin can have long-term effects, particularly for children [1].

A deficiency of vitamin D may cause rickets and its associated skeletal abnormalities, short stature, delayed development, or failure to thrive. The association of vitamin D with biological processes has expanded to include a wide array of health outcomes, such as respiratory infection, asthma, insulin-dependent type 1 diabetes, cardiovascular disease, cancer, and all-cause mortality [2,3,4,5]. Thus, vitamin D deficiency has become an important public health problem despite widespread supplementation and fortification having reduced the incidence of rickets worldwide [6].

To date, several epidemiological studies have provided data on the prevalence of vitamin D deficiency in children and adults around the world [3,7,8,9]. Data estimates of the prevalence of 25(OH) D levels <20 ng/mL—defined as deficient—have suggested that almost 40% of Europeans are vitamin D deficient [10]. The figures for the US and Canada are reported as 47–56% and 37%, respectively [11,12]. A very high prevalence of vitamin D insufficiency, in up to 90% of the infant population, is reported in African and Asian countries [3,13]. Contrary to the general perception, and despite the abundance of sunshine in countries of the middle east, this region suffers from high rates of vitamin D deficiency, varying between 30–90% [14]. Studies from Saudi Arabia have reported high levels of vitamin D insufficiency and a deficiency ranging from 28 to 75% in various age groups [15].

Several factors have been implicated in causing low levels of vitamin D in the region, including low sun exposure and limited outdoor activity due to extreme hot climate, dark skin color, prolonged breastfeeding without vitamin D supplementation, the low calcium content of diets, and lack of policies regarding food fortification with vitamin D [16].

Despite numerous research studies on the status of vitamin D, there is a conspicuous lack of data in infants and children worldwide, together with countries in the middle-eastern region. To the best of our knowledge, this is the first study on vitamin D status in children aged 0–2 years in the Aseer region of Saudi Arabia. This study aims to examine the prevalence of vitamin D deficiency and insufficiency among children in this age range based on the new IOM guidelines [17] and identify the potential factors, including socio-demographics, diet, and sunlight exposure behaviors, that may predispose children to increased risk for vitamin D deficiency or insufficiency. This study data will complement the evidence describing vitamin D status in the present Saudi population of infants and toddlers. We believe that this information is valuable for children’s health as well as the healthcare providers to enable better counseling for parents of young children regarding their need for vitamin D.

## 2. Material and Methods

After obtaining approval from the research ethics committee of King Khalid University, this cross-sectional study was conducted in the pediatric department at the Maternity and Children Hospital, Abha, southern region of Saudi Arabia. The study was conducted from January to November 2021. The sample size of our study has been calculated using a single population proportion test. The estimation was based on the following assumptions: (i) Regional prevalence of vitamin D deficiency in the pediatric age group is 65% (15) (ii) 95% confidence interval (CI), (iii) 5% margin of error, (iv) 80% power and (v) 10% non-response rate. Thus our calculated minimum sample size was 484.Four hundred and eighty-four children (age range, 2–24 months) admitted to the hospital with any acute condition were included in the study. Children with any chronic disease and those who had used any medicine known to affect vitamin D metabolism during the previous 2 months were excluded from the study. The parents were informed about the aim of the study and written informed consent was obtained before the enrollment of the participants. The parents were assured of the confidentiality of all information.

Each parent was interviewed with a questionnaire about the child regarding their age, gender, birth weight, residence, housing status, feeding history, the number of days per week exposed to sunlight, maternal use of vitamin D supplement, and history of rickets in the family. To assess the overall vitamin D status, serum 25-hydroxyvitamin D [25(OH)D] was considered to be the most reliable measure [17]. A blood sample (4 mL) was collected from each child by the ward nurse, and tests were performed in the hospital laboratory. Along with the Serum 25-hydroxyvitamin D assessment, other blood parameters, such as serum calcium, phosphorus, and alkaline phosphatase (ALP), were also measured at the same time. Diasorin’s chemiluminescent immunoassay liaison was used to assess serum 25(OH)D. Serum calcium, phosphorus, and ALP were measured using an Olympus AU 400 chemistry autoanalyzer, serum calcium with a photometric color (Arsenazo method) test, serum inorganic phosphorus by a photometric UV test (phosphomolybdate formation method), and serum ALP with a kinetic color test (*p*-nitrophenyl phosphate substrate in aminomethyl propanol buffer or AMP).

According to the serum 25(OH)D levels, children were categorized into three groups: deficiency (25(OH)D < 20 ng/mL), insufficiency (25(OH)D = 20–30 ng/mL), and sufficiency (25(OH)D > 30 ng/mL) [17,18]. The children were also divided into three age groups: (2–6 months), (7–12 months), and more than 12 months. Furthermore, for the logistic regression analysis, the children were categorized into two age groups (2 to 12 months) and (more than 12 months).

**Statistical analysis:** Data analysis was performed using the SPSS statistical software package (V20, IBM Corp, Armonk, NY, USA). The serum 25(OH)D levels were described using means and standard deviations (SDs). Frequencies and percentages (%) were reported for categorical variables. Mean serum levels of 25(OH) D were compared between groups by using the 2-tailed *t*-test or analysis of variance (ANOVA), where appropriate. The Chi-square test was used to compare non-numerical data. Logistic regression was performed to identify the predictors of the prevalence of vitamin D insufficiency or deficiency. In general, *p* < 0.05 was considered statistically significant.

## 3. Results

Table 1 provides the background characteristics of the toddlers. The mean age of the children was 10.4 ± 6.41 months, with a range of 2–24 months. Males comprised 61%, and infants constituted 63% of the study group. Rural residence was reported in 56.6%, and approximately 72% lived in apartments. The birth weight of 75.6% of children was 3 kg or more, and only 16.1% were exclusively breastfed. Regarding exposure to sunlight, only 30 children (6.2%) were exposed 3 times per week or more. Significant differences were noted in the mean values of serum vitamin D between males and females (*p* = 0.002). The mean serum level of vitamin D was significantly low among infants compared to children aged 12 months and more (*p* = 0.001). The rural residents had higher levels of mean serum vitamin levels as compared to urban dwellers (*p* = 0.012). Children who were exclusively breastfed had lower levels of serum vitamin D compared to those who were fed formula milk. Sunlight exposure 3 times per week or more had a significantly higher level of serum vitamin D (32.43 ng/mL) as compared to those with low exposure (25.22 ng/mL; *p* ≤ 0.001).

Mean serum levels of vitamin D, calcium, alkaline phosphatase, and phosphorus in the total sample are shown in Table 2. The overall mean serum vitamin D level was 25.66 ± 9.11 ng/mL, calcium was 8.82 ± 0.58 mg/dL, ALP was 262 ± 90.36 U/L, and phosphorus was 5.06 ± 0.661 mg/dL. The comparison with standard reference values demonstrated that all overall mean values of the study group were within the normal range. An intergroup comparison showed a significant difference in the levels of vitamin D, calcium, and alkaline phosphate (*p* < 0.001). The highest level of alkaline phosphatase was observed in the vitamin D insufficiency and deficiency group (*p* < 0.001).

**Figure 1:**
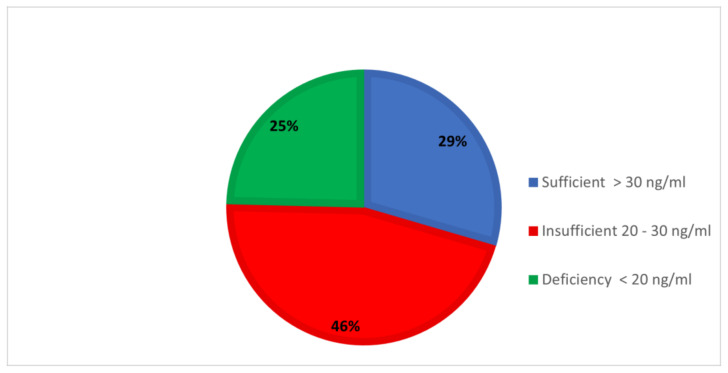
Distribution of vitamin D levels.

Figure 1 shows the distribution of the study group according to the level of serum vitamin D. About 30% of the toddlers had sufficient levels of vitamin D, whereas 45.9% had insufficient levels, and one-fourth (24.6%) showed deficiency. Thus, an overall deficiency of vitamin D was observed in 70.5% of the children.

Table 3 describes the comparison of serum vitamin D levels with the background characteristics of the study group. Males (28.1%) were found to be more deficient than females (19%; *p* = 0.032). In the infant age, insufficiency and deficiency were significantly higher (*p* < 0.001) as compared to children aged 12–24 months. Regarding rural-urban differences, the proportion of deficiency was almost equal; however, insufficiency was significantly higher in the urban group (54.3%; *p* < 0.001). The deficiency was significantly higher in apartment dwellers. Comparing vitamin D levels by birth weight revealed significantly higher insufficiency in those with a birth weight of more than 3 kg. History of breastfeeding showed a significantly higher percentage of deficiency (41%) and insufficiency (44.9%) in the breastfed infants compared with infants who were fed formula milk either complementary to breast milk or by itself (*p* < 0.001). Significantly higher levels of deficiency were found in children who had low sunlight exposure (26%) as compared to those with adequate sunlight exposure (3.3%; *p* < 0.001).

The independent predictors of vitamin D deficiency, as revealed by logistic regression analysis, are presented in Table 4. Multivariate regression analysis was performed using only those variables that were found significant in the univariate analysis. The children aged 2–12 months (infants) were more likely to be vitamin deficient compared to the children aged 12 months and above. The children who lived in urban areas had a threefold increased risk of vitamin deficiency compared to the children living in rural areas (aOR = 3.0, 95% CI 1.78–5.08). The children who had been exposed to sunlight for less than 3 days per week experienced an increased risk of developing vitamin D deficiency (aOR = 4.17, 95% CI 2.04–10.88). Children who had received only breast milk were more than two times more likely to be vitamin D deficient (aOR = 2.42, 95% CI 1.12–5.23) compared to their counterparts.

## 4. Discussion

Infancy, childhood, and puberty are periods of rapid growth. During these stages, vitamin D is vital for skeleton formation, and its deficiency can lead to skeletal and extra-skeletal abnormalities. This study determined the prevalence of vitamin D deficiency and insufficiency in Saudi infants and toddlers and its associated factors.

In this research, less than a third of the toddlers had sufficient levels of vitamin D, whereas one-fourth showed deficiency. The combined prevalence of insufficiency and deficiency reached a high proportion, which supports the data on the global endemicity of vitamin D deficiency.

High levels of Vitamin D deficiency have been globally reported in various studies. African [3] and Asian [13] countries report higher deficiency rate than Europe [10], America [11] and Canada [12]. Middle eastern countries have also shown a high prevalence of Vitamin D deficiency despite the sunny climate throughout the year [14,15,16].

Studies from Saudi Arabia have reported high levels of vitamin D insufficiency and deficiency, ranging from 28–75% in various age groups [17,18,19,20].

We calculated the mean serum values of all studied biomarkers for our study population and found them within the normal range of standard reference values [21]. However, when we compared the values of calcium, phosphorus, and alkaline phosphatase in relation to the vitamin D levels, we found significant inter-group differences. The lowest level of calcium was observed in the group with severe vitamin D deficiency. This finding agrees with the established relation of deficient calcium levels with vitamin D deficiency. Moreover, active vitamin D facilitates the absorption of calcium and phosphorous from the gut, and consequently, its deficiency reduces calcium and phosphate absorption and causes low levels [22,23]. A significant relationship was found between the highest levels of alkaline phosphatase and severe vitamin D deficiency. Although in current clinical practice, alkaline phosphatase is used as a marker of vitamin D deficiency due to the low cost of the test, its results could be misleading unless used in conjunction with other biomarkers such as calcium, phosphorous, and, most importantly, vitamin D, and its use as an independent biomarker of vitamin D deficiency is not recommended [24].

We observed that males, infants, urban dwellers, children who were fed only breast milk, and those with poor sun exposure had a significantly lower level of mean serum vitamin levels and a higher prevalence of vitamin D deficiency. The findings of males having lower levels of vitamin D and a higher rate of vitamin D deficiency are different from other studies in the middle east that report a higher prevalence among females [25,26]. This variation may be due to differences in the study population and different settings, such as in our research, which investigated toddlers, whereas the other studies analyzed adolescents and adult females. Another reason for these differences may be the study setting, which was hospital-based in our case, rather than in the community.

Urban dwellers had lower levels of mean serum vitamin D as compared to rural dwellers. A similar urban-rural difference was observed in India and Malaysia [27,28,29]. Urban living and sunlight exposure are related. In urban areas, there is a lack of space and overcrowded tenements that prevent direct sunlight from reaching inside most parts of urban regions and gives limited scope for outdoor activities among children. Considering that vitamin D is primarily made in the skin after exposure to ultraviolet radiation (UVR), low sunlight exposure reflects the absence of its most important source [3,5]. Today, most children tend to spend more time indoors than outdoors. Excessive time spent indoors reduces sun exposure and, therefore, leads to decreased vitamin D synthesis. In countries such as India and China, air pollution has contributed to high haze scores, which reduce the number of solar UVB rays reaching the ground, hampering vitamin D synthesis [30,31,32]. The extreme discomfort of the middle-eastern sun also keeps children away from the sunlight. This issue particularly holds true for toddlers, who are primarily at home and neither receive sufficient exposure through school nor during play outdoors.

In this study, an age of 2–12 months (infants), urban-dwelling, low exposure to sunlight, and only feeding with breast milk were identified as independent predictors of vitamin D deficiency. In the middle east, low sun exposure and limited outdoor activity due to extreme hot climate, dark skin color, and prolonged breastfeeding in the absence of vitamin D supplementation may contribute to vitamin D deficiency in the infant population. These factors have been implicated for such deficiency in earlier studies from this region. Increasing age, female gender, high weight, or body mass index (BMI), low physical activity, low intake of calcium or vitamin D supplements, concealed clothing, low sun exposure duration, winter season, lower education or socio-economic status, and urban residence have been consistently identified as predictors of low 25(OH)D levels across the lifecycle in this region [15,16,17,18,19,20]. Because breastmilk has poor vitamin D content, breastfeeding without vitamin D supplementation is an important risk factor for its deficiency in this age group. This issue has been reported in research from India, South Korea, and South Africa [33,34,35,36].

The results of this study have important public health implications. The significance of sunlight exposure, vitamin D supplementation, and breastfeeding need to be emphasized for the mothers of young children as well as for the public. Vitamin D-rich supplements such as cod liver oil must not only be advocated but also incorporated into the national food fortification and supplementation initiatives. Prior studies have revealed poor rates of breastfeeding in Saudi population [37], hence health messages on exclusive breastfeeding need to be reinforced.

## 5. Strengths and Limitations of the Study

To our knowledge, this is the first study to describe the status of vitamin D levels in children up to 2 years of age in Saudi Arabia. Due to its cross-sectional nature, causations were not able to be analyzed. We also acknowledge the limitation that we assessed sunlight exposure by using a questionnaire, and objective measures such as polysulfone badges or measuring haze scores were not used. In addition, the timing and duration of sun exposure were not determined. In this study, female participants were less than males. It had been more female children included in the study, the results might look different. Another important limitation of this work is that it was performed in only one children’s hospital; hence, a multicenter national study is required to validate/generalize our results.

## 6. Conclusions

This research revealed a high prevalence of vitamin D deficiency among children aged up to two years in the southwestern region of Saudi Arabia. Young age group <12 months (infants), urban dwellers, breastfed only, and exposure to sunlight for less than 3 days per week were identified as independent risk factors for vitamin D deficiency. The results of this study demand an enhanced awareness to ensure adequate levels of vitamin D for better health of the children in this region of Saudi Arabia. Our results have important public health implications because vitamin D deficiency is clearly a problem in this community among very young children; exposure to sunlight appears to be an appropriate approach for decreasing this issue’s magnitude. Information regarding the importance of sunlight exposure for young children should be emphasized to mothers and the general community.

## Figures and Tables

**Table 1:** Background characteristics and serum 25 (OH) D level among the children.

Background Characteristics	n%	Mean & SD ng/mL	*p*-Value
**Gender**			
Male	295 61.0%	24.64	0.002
Female	189 39.0%	27.26	
**Age group**			
2–6 months	160 33.1%	24.3	0.001
7–12 months	144 29.8%	24.7	
>12 months	180 37.1%	27.7	
**Residence**			
Rural	274 56.6%	26.57	0.012
Urban	210 43.4%	24.48	
**Housing status**			
Villa	136 28.1%	26.62	0.149
Apartment	384 71.9%	25.29	
**Birth weight**			
<3 kg	118 24.4%	24.45	0.097
≥3 kg	366 75.6%	26.05	
**Feeding history**			
Breast milk only	78 16.1%	20.92	<0.001
Breast milk & formula milk	194 40.1%	25.54	
Formula milk only	221 43.8%	27.52	
**Weekly exposure to sunlight**			
<3 times/week	454 93.8%	25.22	<0.001
≥3 times/week	30 6.2%	32.43	
**Family history of Rickets**			
Yes	98 20.2%	25.52	0.858
No	386 79.8%	25.70	
**Mother uses vitamin D** **supplements**			
Yes	62 12.8%	26.56	0.566
No	422 87.2%	25.60	

**Table 2:** Comparison of biochemical markers by vitamin D status.

Biochemical Markers	Reference	Overall *n* = 484	Sufficient *n* = 143	Insufficiency *n* = 222	Deficiency *n*= 119	F Value & df	*p* Value
Vitamin D (ng/mL)	20–50	25.66 ± 9.11	35.89 ± 4.59	25.63 ± 3.0	13.43 ± 4.46	1071.7 (2)	<0.001
Calcium (mg/dL)	8.80–10.80	8.82 ± 0.58	9.05 ± 0.48	8.85 ± 0.47	8.50 ± 0.71	33.17 (2)	<0.001
ALP (U/L)	100–550	262 ± 90.36	240.45 ± 87.81	260.48 ± 79.66	291.16 ± 104.1	10.7 (2)	<0.001
Phosphorous (mg/dL)	4.0–7.0	5.06 ± 0.661	4.99 ± 0.64	5.07 ± 0.59	5.14 ± 0.77	1.68 (2)	NS (0.187)

**Table 3:** Comparison of serum vitamin D levels with the background characteristics.

Variables	Status of Vitamin D			
	Deficiency n%	Insufficiency n%	Sufficiency n%	*p* Value
**Gender**				
Male	83 (28.1)	135 (45.8)	77 (26.1)	0.032
Female	36 (19.0)	87 (46.0)	66 (34.9)	
**Age**				
2–12 months (infant)	85 (28.0)	148 (48.7)	71 (23.4)	<0.001
More than 12 months	34 (18.9)	74 (41.1)	72 (40.0)	
**Residence**				
Rural	66 (24.1)	108 (39.4)	100 (36.5)	<0.001
Urban	53 (25.2)	114 (54.3)	43 (20.5)	
**Housing status**				
Villa	21 (15.4)	79 (58.1)	36 (20.5)	0.001
Apartment	98 (28.2)	143 (41.1)	107 (30.7)	
**Birth weight**				
<3 kg	39 (33.1)	43 (36.4)	36 (30.5)	0.02
≥3 kg	80 (21.9)	179 (48.9)	107 (29.2)	
**Feeding history**				
Breast milk only	32 (41.0)	35 (44.9)	11 (14.1)	<0.001
Breast milk & formula milk	52 (26.8)	80 (41.2)	62 (32.0)	
Formula milk only	35 (16.5)	107 (50.5)	70 (33.0)	
**Exposure to sunlight**				
<3 times/week	118 (26.0)	211 (46.5)	125 (27.5)	<0.001
≥3 times/week	01 (3.3)	11 (36.7)	18 (60.0)	
**Family history of Rickets**				
Yes	22 (22.4)	51 (52.0)	25 (25.5)	0.382
No	97 (25.1)	171 (44.3)	118 (30.6)	
**Mother uses vitamin D supplements**				
Yes	12 (37.5)	14 (43.8)	06 (18.8)	0.537
No	113 (25.0)	208 (46.0)	131 (29.0)	

**Table 4:** Multiple logistic regression model for determinants of vitamin deficiency.

Variables	β	aOR *	*p*-Value	95% Confidence Interval (CI)
Age				
>12 months				Reference
2–12 months	0.540	1.71	0.017	1.10–2.67
Residence				
Rural				Reference
Urban	1.101	3.0	<0.001	1.78–5.08
Exposure to sunlight				
≥3 days/week				Reference
<3 days/week	1.551	4.71	<0.001	2.04–10.88
Feeding				
Formula feeding				Reference
Breast milk & formula feeding		1.09	0.703	0.684–1.75
Only breast milk	0.887	2.42	0.024	1.12–5.23
Constant	1.554		<0.001	

aOR * = Adjusted odds ratio.

## Data Availability

The datasets used and analyzed in the current study are available from the corresponding author on reasonable request.
